# The Structure and Effectiveness of Health Systems: Exploring the Impact of System Integration in Rural China

**DOI:** 10.5334/ijic.2197

**Published:** 2016-08-12

**Authors:** Xin Wang, Stephen Birch, Huifen Ma, Weiming Zhu, Qingyue Meng

**Affiliations:** 1 School of Medicine and Health Management (formerly known as Center for Health Management and Policy), Shandong University, Jinan, China; 2 Department of Clinical Epidemiology & Biostatistics, McMaster University, Hamilton, Canada; 3 China Center for Health Development Studies, Peking University, Beijing, China

**Keywords:** county health system, integration, social network analysis

## Abstract

**Introduction:**

Facing the challenges of aging populations, increasing chronic diseases prevalence and health system fragmentation, there have been several pilots of integrated health systems in China. But little is known about their structure, mechanism and effectiveness. The aim of this paper is to analyze health system integration and develop recommendations for achieving integration.

**Method:**

Huangzhong and Hualong counties in Qinghai province were studied as study sites, with only Huangzhong having implemented health system integration. Questionnaires, interviews, and health insurance records were sources of data. Social network analysis was employed to analyze integration, through structure measurement and effectiveness evaluation.

**Results:**

Health system integration in Huangzhong is higher than in Hualong, so is system effectiveness. The patient referral network in Hualong has more “leapfrog” referrals. The information sharing networks in both counties are larger than the other types of networks. The average distance in the joint training network of Huangzhong is less than in Hualong. Meanwhile, there are deficiencies common to both systems.

**Conclusion:**

Both county health systems have strengths and limitations regarding system integration. The use of medical consortia in Huangzhong has contributed to system effectiveness. Future research might consider alternative more context specific models of health system integration.

## Introduction

The health system in China is facing considerable challenges associated with an aging population and the increasing prevalence of chronic conditions among the older population together with increases in health care utilization and cost. Forecasts by the National Aging Office, suggest that between 2001 and 2020 the Chinese population aged 60 and older will increase by an average of 5.96 million per year and will reach over 17% of the total population by 2020 [1]. According to the National Health Service Surveys of 1998 and 2008, new cases of chronic diseases increased on average by 10 million each year over that period [2]. This epidemiological transformation emphasizes the need to pay more attention to health prevention, rehabilitation and case management. At the same time, rapid urbanization brings increasing demands for health improvements. The integration of health care planning, management and delivery provides a potential strategy for addressing these challenges.

Over the past 30 years, the relatively integrated three-tier network of treatment and referral built during the era of the planned economy in China has been broken. With the development of a market economy, decentralization and the reduction in financial allocations from Central government contributed to the collapse of the integrated system [3]. Nowadays, health care in China suffers from fragmentation and lack of coordination between different health care institutions, and community care institutions do not serve as gatekeepers to other institutions in the system [4]. Moreover, following health care reforms in 2009, the national level of enrollment coverage in basic health insurance in now is more than 95%, patient reimbursement rates are averagely 70%, both of them resulted in increased demands for health care and less out of pocket costs to patients in higher-level institutions than before. For example the percentage nationally of patients hospitalized increased from 6.8% in 2007 to 9.0% in 2012 [5]. With less out of pocket costs, more patients went to tertiary hospitals directly, which led to cost escalation of the whole health care system [2]. In the absence of cooperation both vertical and horizontal, among health care institutions, the fragmented health system struggles to deliver comprehensive and high-quality health care interventions for patients. A more integrated system offers the potential to meet the needs of communities in ways that are more effective and efficient.

There have been several pilot projects of integrated health systems in China in the last decade [6,7,8,9], aimed at providing integrated and high-quality care at low cost. In practice, most of the projects focused on vertical integration among hospitals [10]. Existing research in China has been focused on theoretical explorations and the experiences of other countries. A few evaluation researches were reported. Most of them were qualitative or were limited to a few quantitative indicators, such as bed occupancy rates, revenues, number of joint training programs [8,9,11,12]. Hence, little is known about integration of the existing health systems, how the systems function and whether they are effective.

In general, health care systems worldwide have been designed primarily to deal with single, acute, and short-term illnesses [13,14,15], however emerging from the epidemiological transition, health systems in developed countries also suffered fragmentation [16,17]. Over the last decade, integration has been suggested as one possible strategy to promote coordinated health care delivery and improve quality of care at lower cost [18,19,20] in developed countries. Many have launched integration pilots [21,22,23,24,25,26,27] and researchers have found that integrating local health services can achieve target outcomes more easily with less investment.

For studies of integrated care, social network analysis has been seen by many as a valuable method [28,29,30,31,32,33]. Different from traditional analytic methods, which pay attention to the characteristics of individual actors, network analysis focuses on the relationships among actors [29]. Therefore, it is helpful to shed light on the interrelationships among institutions in a health system, to identify the integrated structures of the systems, and to assess their effectiveness. For instance, whole network analysis has been used to understand the structure of a health promotion network in Canada, and to identify the types of connections shared by network members [33].

The aim of this paper is to analyze the integration of the health care system in rural China using two contrasting cases from the Qinghai province in western China. The hypothesis is that the health system in the county, which builds medical consortia, is more integrated and effective than in another county. A methodology for describing and evaluating health systems will be presented, and policy recommendations developed for health care system integration in China. To the best of our knowledge, this is the first paper to show the structure, operation and effectiveness of a county health system using social network analysis in China.

## Study Setting

### Context of Qinghai province and two counties

Qinghai province is in western China with a population of 5.78 million in 2013. Per capita GDP in 2014 was $6,252, ranking 21st among 31 provinces and municipalities in China. In addition to limited economic resources, there is a severe shortage of other health resources, especially the qualified workforce. The development of the health delivery system in Qinghai is less advanced than most other provinces with the need to improve the efficiency of resource utilization and service delivery. As the first province working on integration at provincial level, Qinghai Province implemented two policies in 2013: the village or township doctor as the first point of contact and gatekeeper to other institutions (referrals to higher level institutions for treatment and lower level institutions for rehabilitation). In addition, considerable resources were provided for developing information technology to promote the policy implementation. As a typical western province with low GDP, the practice of health system reform in Qinghai province might provide evidence for policy developments in other provinces.

As the only county with reform of health system structure, Huangzhong provided the focus for this study. In order to show the effect on integration and effectiveness of the health system by comparison, Hualong was adopted as the second county, because of two reasons: (1) they had similar health resources and implemented four important health reforms (see the bottom four lines in Table 1); (2) Hualong had no adjustments to the health system structure, and the two provincial policies were not implemented comprehensively.

**Table 1:** Basic information of Huangzhong and Hualong (2013).

Indicators	Huangzhong County	Hualong County

Population (thousands)	480.6	276.6
GDP per capita (RMB)	31484	16537.7
Income per capita (RMB)	8064	5854
Average life expectancy at birth (years)	71.3	70
No. of hospital beds (per thousand population)	2.34	2.40
No. of practitioners/assistant practitioners (per thousand population)	0.96	0.96
No. of staff in professional public health institutions (per thousand population)	0.21	0.21
No. of outpatient admission (thousand)	270. 0	266.1
No. of inpatient admission (thousand)	29.0	24.9
% population enrolled in NCMS*	99.68	99.87
“Equalization of basic public health services” Project**	From 2009	From 2009
Reform of compensation mechanism in county hospital	Form June, 2012	From April, 2014
Zero-markup of drug	From September, 2010	From January, 2011

* NCMS is short for New Cooperative Medical Insurance System, the insurance for residence in rural China. ** “Equalization of basic public health services” is a project, which delivers 13 preventive health services for all people in order to reduce health disparities.

### Health system reform practice in two counties

Huangzhong implemented health system reform in September 2013, aimed at promoting cooperation among institutions at different levels and controlling the rate of increase in medical costs. The health care institutions of the county were grouped into three consortia based on the levels of institutions and their location. Each consortium consists of one county hospital, a few township health centers and many village health stations. The county hospital, as a leader, is responsible for the management of general affairs in the consortia, including organizing routine meetings, sharing health resources, conducting joint training, collecting information, receiving patients referred from other institutions, and so on. In contrast, township health centers comply with the management of the county hospital and are expected to refer patients to the county hospitals.

Governance of medical consortia in Huangzhong is at two levels. At the county level, a leadership group is responsible for construction planning, operating instructions, quality control and assessments, and dealing with problems and challenges common to all consortia in the county. Members of the lead group are leaders of different government departments, including the health, finance, human resources and social security departments, together with the development and reform commission. A small number of professional officers of the county health department are responsible for the daily work of the lead group. There is a council in each consortium, which is responsible for negotiating internal cooperation, such as development planning and resource sharing.

No reforms involving reorganization and integration of the health delivery system has taken place in Hualong, but other provincial health reforms (Table 1) are similar to Huangzhong. It remains a traditional rural three-level health system, with no specific requirement of cooperation in the system. The health department and family planning commission are jointly responsible for all work, including development planning, cooperation agreements, monitoring implementation and evaluating effectiveness. Only one professional officer of the county health department deals with daily work related to cooperation among institutions within the county.

## Method

Network analysis is employed to illustrate the structure of existing relationships among institutions [29] in each health system and to measure and compare integration among systems.

### Data sources, collection and analysis

The network boundary of this research is county jurisdictions. Each county is described by its context, participation and level of health system reform, governance and integration effectiveness.

The survey was conducted in August and September, 2014. To ensure a greater response rate we received support from the local health departments and went to each study institution with departmental staff. All institutions at county level are included in the analysis along with 3 township health centers and 6 village health stations in each county. Distance among institutions is an important factor of their connections. Township health centers within the similar distance to county hospitals are supposed to show similar relationship with them. Therefore, not all township health centers were included in the study. Stratified sampling was used based on their distances to county hospitals. The distances of three township health centers to Huangzhong county hospital are about 5km, 10km and 15km. While in Hualong, the distances are about 20km, 30km and 40km, since Hualong is larger than Huangzhong. 6 village health stations were adopted in each county by stratified sampling, according to their distances to the township health centers included in the study.

Data on relationships within the network were collected using a modified version of Provan’s instrument [35] (see Appendix 1). This institutional questionnaire was completed through face to face interviews (30 minutes on average) with the key informant in each institution. The key informant was identified by the head of institution as most knowledgeable about interrelationships with other institutions. The interview asked about services provided, growth of the institution (staff, facilities, etc), positive impacts and barriers of collaboration with other institutions in the network.

Effectiveness data were collected by questionnaires completed by doctors and patients, and inpatient data from the counties’ health insurance reimbursement systems. The most authoritative doctors from each clinical department in each institution, as identified by the heads of institutions, completed questionnaires about their behaviors related to four types of relationships (referral, population health information sharing, patient medical information sharing, and joint training). As the numbers of departments vary across institutions, the numbers of doctors interviewed are different in the two counties. Randomly selected outpatients and inpatients completed questionnaires, which asked about satisfaction with services involving institutions at different levels.

In addition, all questionnaires were completed under the guidance of trained investigators and all interviews were conducted by researchers. The number of interviewees is shown in Table 2, and the response rate is 100%.

**Table 2:** Number of interviewees in each county.

	Huangzhong	Hualong

Institution leaders	15	14
Doctors	39	27
Patients	28	16

Information on between institution relationships was analyzed using UCINET 6, software for social network analysis. Unconfirmed connections were included in the analysis, which meant that it wasn’t necessary for both partners indicating connections with each other. Other research has focused on the more reliable “confirmed connections”, as indicated by a mutual recognition of the connection [34]. More recently it has been suggested that only using confirmed connections may underestimate the collaboration in networks [35]. Therefore, unconfirmed connections may not be discounted, especially in exploratory studies [33]. Interviews were transcribed word by word, and thematic analysis was conducted by MAXQDA 11.

### Indicators and measurements

**Figure 1:**
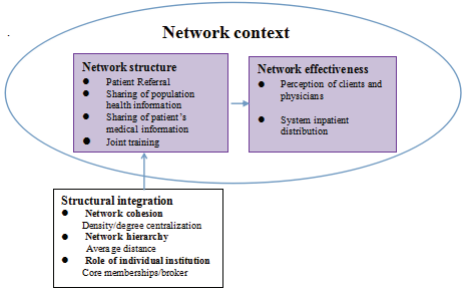
Measurement and indicators of network effectiveness evaluation [36].

The network effectiveness evaluation model, an adaptation of Provan’s preliminary model [36] is presented in Fig. 1.

The system structure was measured by five indicators from three dimensions. Density and degree of centralization are two of the most commonly used network indicators [29]. Density reflects the number of connections in a network, and is expressed as a percentage of total possible number of connections [37]. Degree of centralization of a network quantifies the range or variability of the individual degree centrality, which refers to the structure of power and control in a network [38]. Average distance is important because the health system network in this study is inherently hierarchical. The average distance is measured by the average geodesic distance from a village health station to institutions out of county in the referral network and to county-level institutions in other networks. Core/periphery class memberships and brokerage roles reflect the roles institutions serve in the network. A network has a core/periphery structure if it can be partitioned into two sets: a core and a periphery. Core members are densely tied to each other and also have connections with periphery members. Brokerage roles [39] examine ego’s acting as an agent in relations among its neighborhood, where the actor lies on the direct path between two other actors. In this study, institutions within the county jurisdiction are classified as one group and the other institutions as another group. We focus on two of the five broker roles [40], coordinator within the county group and representative for the county group for their relationship with another group.

Provan proposed three levels for Network evaluation: community [36], network [41], and organization/participant [33,42]. In this paper, we focus on participant level and network level, from the perspective of staff and patients. Six indicators (see Table 4 below), related to four types of integration (see Fig. 1), were measured by doctors’ behaviors and patients’ satisfaction. Effectiveness at network level was evaluated by change of inpatients distribution. Patient satisfaction was measured using a 5-point Likert scale from 1 (strongly satisfied) to 5 (strongly dissatisfied). Patient’s distribution was measured by the percentage of inpatients hospitalized in town-level institutions, county-level institutions and institutions out of county. We collected the number of inpatient admissions for which the hospital was reimbursed for an 18-month period (January, 2014 to June, 2015), nine months before and nine months after health system reform. The change was compared before and after the health system reform in each county and then it was compared between two counties.

**Table 3:** Network integration measurement.

Indicator	Referral network		Population health information sharing network		Patient’s health information sharing network		Joint training network	
			
	Huangzhong	Hualong	Huangzhong	Hualong	Huangzhong	Hualong	Huangzhong	Hualong

Density	0.095	0.117	0.110	0.093	0.423	0.424	0.123	0.102
Degree centralization	0.220	0.428	0.203	0.160	0.189	0.145	0.546	0.693
Avg Distance	2.667	1.750	2.50	2.00	—	—	1.75	3.0
Core memberships	CH1/CH3/THC1/THC2/THC3	PH1/CH1/THC1/THC2/THC3	CDC/MCH/THC1/THC2/THC3	CHD/MCH/THC1/THC2/THC3	CH1/CH2/CH3/MCH/THC1/THC2/THC3	CH1/CH2/MCH/THC1/THC2/THC3	CH1/MCH/THC1/THC2/V22	CH1/MCH/THC1/THC2/THC3
Brokerage roles	Coordinator: CH1/THC1/THC2/THC3 Representative: THC2/THC3	Coordinator: CH1/THC1/THC2/THC3 Representative: CH1/THC2	—	—	—	—	Coordinator: CH1/CH3/MCH/THC1/THC2/THC3 Representative: CH1/MCH	Coordinator: CH1/MCH/CDC/THC1/THC2/THC3 Representative: MCH/THC2

**Table 4:** Participants’ experience of network effectiveness.

Dimension	Indicator	Results	
		
		Huangzhong	Hualong

Referral network	Percentage of doctors who referred patients	97.44	77.78
	Patient satisfaction with referral	71.43	63.75%
Sharing of basic health information for population	Percentage of doctors who used e-health record	64.10	40.74
Sharing of medical information for patients	Percentage of doctors who recognize patients’ medical information from same-level institutions	39.40	47.62
Joint training network	Percentage of doctors who went to upper institutions for training (in 2013)	84.62	59.26
	Patient satisfaction with doctors’ ability	89.29	87.5

## Results

Relationships are plotted using NETDRAW, which is an embedded function of UCINET. Nodes indicate institutions, lines show connections and arrows illustrate the direction of relationships. The institutions, which were nominated by surveyed institutions and located outside the two counties, were adopted in the figures. The figures are organized by levels of institutions, and the shape of a node indicates the type of institution. Nodes colored black indicate core members in a network.

### Referral network

**Figure 2:**
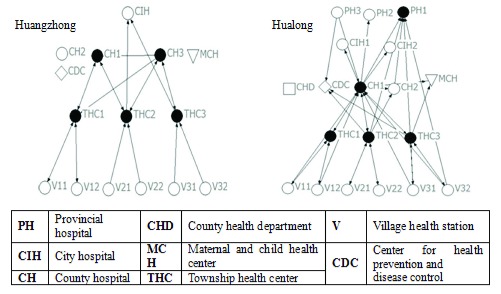
Referral Networks.

Figure 2 and Table 3 indicate that, compared to Huangzhong, there are more out-of-county institutions involved in the referral network of Hualong. As more referrals flow to County Hospital 1, the general cohesion of the Hualong network is higher. Shorter average distance from a village health station to institutions out of county implies more ‘leapfrog’ referrals in Hualong, as shown by Fig. 2. Similarly, township health centers in both countries are core members and serve as coordinators inside the county. Two county hospitals have core roles and receive most referrals in Huangzhong, while Provincial Hospital 1 gets referrals from more institutions than County Hospital 2 in Hualong. In summary, based on the referral refinement criteria of Qinghai province [43], the referral network structure in Huangzhong is more normalized and balanced than in Hualong.

### Information sharing network

Information is an important resource and sharing information is a necessary step for other types of integration [41,44]. In this paper, population health information includes e-health records and disease prevalence reporting. Meanwhile, patient’s medical information consists of laboratory results, medical images and reports.

#### Sharing of population health information

**Figure 3:**
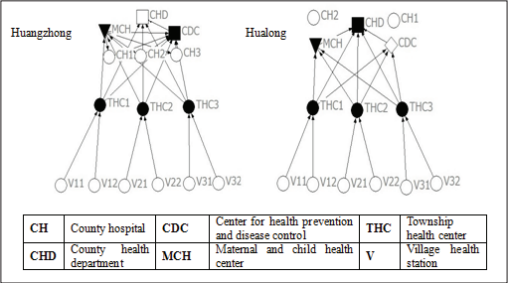
Population health information sharing networks.

As illustrated by Fig. 3 and Table 3, both networks are vertically unidirectional (from lower levels institutions to upper levels). Two county hospitals in Hualong do not share e-health records with the other institutions. Average distance from a village health station to accessible county-level institutions of Hualong is shorter than in Huangzhong, which means basic health information of the population transfers faster across hierarchies in Hualong. The county health department of Hualong collects and stores information from all information in the county, but in Huangzhong county, the county health department does not collect information from the maternal and children’s hospital.

#### Sharing of patient medical information

**Figure 4:**
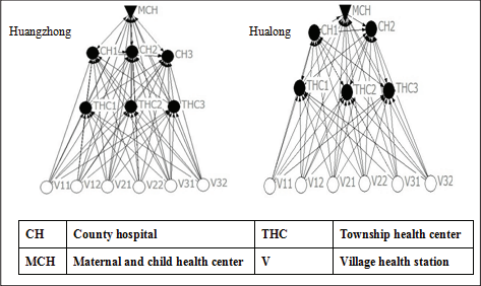
Patient’s medical information sharing networks.

As shown in Fig. 4 and Table 3, the networks for sharing patient’s medical information in each county are “perfect”, with large size, high density and high cohesion. All institutions recognize patients’ medical information from upper-level institutions and institutions at the county level recognize each others’ information. However, there is zero-connection among township health centers in both counties. The difference between the two networks is that county hospitals in Hualong recognize information from township health centers. This is not the case in Huangzhong.

### Joint training network

**Figure 5:**
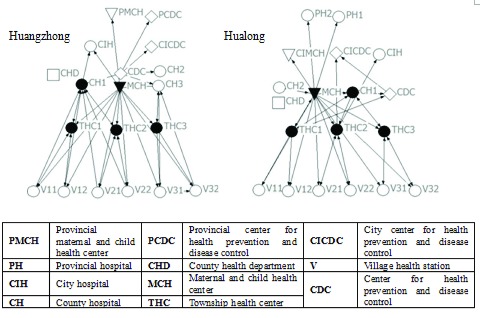
Joint Training Networks.

The joint training networks (see Figure 5) in both counties are the same size and cover a wide range of institutions, which provides the foundation for comprehensive development of doctors’ skills and multiple-institution cooperation. The higher degree of centralization of the Hualong network reveals considerable service delivery pressure of the maternal and children’s health center and county hospital 1. The average distance from a village health station to accessible county-level institutions in Huangzhong County is shorter than in Hualong County, which means it is easier for the lowest level institutions to get direct training from county hospitals. Centers for disease control and prevention in both networks deliver less training than the maternal and children’s health centers or county health departments. Regarding broker analysis, the key institution in Hualong is county hospital 1, but maternal and child health center and center for disease control and prevention are the key institutions in Huangzhong.

### Network effectiveness

Table 4 compares participants’ experiences of network effectiveness in the two counties. Patient satisfaction is defined as strongly satisfied and satisfied, namely 1 and 2 in the 5-point Likert scale. This table illustrates that almost all indicator scores are higher in Huangzhong than in Hualong, especially the percentage of doctors who referred patients and the percentage of doctors who went to upper institutions for training. Meanwhile, the higher percentage of doctors who shared patient medical information with same-level institutions found in Hualong is in accordance with mapping (Fig. 4) and measurement (Table 3) above.

Regarding effectiveness at the network level, the percentage of inpatients going to upper county-level institutions in Huangzhong decreased by 3.53% between January, 2014 and June, 2015.(see Table 5). This shift in inpatient activity towards the other levels (town and county) over this period shows that more inpatients received appropriate treatment within the county at lower per patient cost [45]. However, the 1.5% increase in inpatients at the upper county level and the 11.0% decrease in inpatients at the town level in Hualong imply a reduction of township health centers’ service delivery and a reduction of county health system effectiveness.

**Table 5:** Percentages of inpatients in different level institutions (%).

Level of institution	Huangzhong			Hualong		
		
	2014.1–2014.9	2014.10–2014.6	change	2014.1–2014.9	2014.10–2014.6	change

Town-level	23.86	24.19	0.33	62.18	51. I8	–11.0
County-level	30.05	33.25	3.20	14.63	24.13	9.50
Upper county-level	46.09	42.56	–3.53	23.19	24.69	1.50

## Discussions

The study has demonstrated how two county health systems in rural China are structured, and how effective the two systems perform under these structures. Specially, it identifies four types of relationships among institutions, which presents opportunities and constraints for health system integration. The impact of those relationships on service-delivery effectiveness at the participant level is evaluated from the perspective of care provider and care recipient.

Network analysis, the method used to describe and assess county health systems, is not without problems or shortcomings. The primary benefit is that it allows network members to get a clear picture of connected institutions and the roles of individual institutions. However, it provides a snapshot at one point in time. Only when conducted regularly, can network analysis assess the dynamic nature of system development, including the evolution of contextualization, structure, and effectiveness of health system.

Through mapping and measurement of the system structure, it is shown that the health system in Huangzhong is more integrated than that in Hualong. The notable similarity in both counties is in the area of information-sharing. The sharing networks for patient medical information are the most dense and most cohesive among the four types of networks. In 2008, Qinghai provincial health department issued a policy [46], which required all health institutions to recognize patient medical information from upper-level and same level institutions. Therefore, policies on integrated care matter, as has been supported by findings in six European countries [47]. The sharing network of population health information in neither county has the greatest number of ties in four types of networks, which is not in accordance with previous research in developed countries [48,49]. According to interviews with the heads of institutions and doctors, there are two reasons for the difference. First, health information systems are less developed in China than in many high-income counties. Second, many staff in the surveyed institutions aren’t familiar with information systems, with contacts with other institutions being by telephone or paper-documents. A high level of shared information is a good indicator of network potential because community capacity generally begins with it. [49,50]. Therefore, further development of information sharing networks may bring more collaboration in health systems [51] by solidifying other types of integration. For example, bidirectional sharing of patient medical records is the basis for improved patient referral among different institutions.

Considerable structural differences were found between the two counties’ systems. System structures of Huangzhong, both referral and joint training, have clearer divisions of responsibilities and more cooperation than in Hualong. The composition of small groups is in accordance to the boundaries of the three consortia, which implies that the development of consortia had the effect of promoting cooperation and integration among institutions in the same consortia. However, there is an obvious deficiency in Huangzhong: the absence of the maternal and children’s health centers in the three consortia results in no connections in the referral network and less connection than Hualong in the joint training network. More importantly, this implies fragmentation of preventive care and maternal and child care, which runs in the opposite direction with integrated care. In parallel, the health system of Hualong also presents limitations. The township health centers and village health stations do not serve as gatekeepers in the referral network of Hualong, which may result in outflows of patients to higher level institutions and increases of health care costs.

In role analysis from the perspective of ego, township health centers in both networks perform as coordinators between village health station and institutions at the county level, in both referral and joint training networks. However, recent research found less treatment services delivered and low quality of preventive services in township health centers after the implementation of the “Equalization of Basic Public Health Service Project”, in which 13 preventive health services were delivered for the whole population in order to reduce health disparities [52,53]. The project resulted in doctors in the village health stations and township health centers spending more time on preventive care than before, so it resulted in fewer time on treatment service and lower quality care. In order to promote the coordination role of the township health centers, measures are required to improve the capacity of these health centers. A significant finding in the analysis of core/periphery class memberships is that there are fewer county-level institutions in Hualong serving as core members, which means a relatively strong dependence on institutions located outside the county and the development of a monopoly position by county hospital 1. Following West. (1999), compared to cohesive groups or cliques, the central actor in a highly centralized network has a disadvantage in ensuring that all members follow suit and that members maintain their identity and sense of belonging [51]. Hence, it is necessary to promote the development of other county-level institutions in Hualong for sustainable and coordinated health care system development.

With almost all indicators of effectiveness being higher in Huangzhong than in Hualong, the effectiveness results are consistent with integration measurement by social network analysis. Since Huangzhong implemented two provincial policies well along with consortia building, we could not tell the influencing facator of higher effectiveness is gatekeeping program or the establishment of consortia. But some key informants implied in the interviews that consortia provided organizational basis for gatekeeping program and patient referral. Therefore, the establishment of consortia in Huangzhong was instrumental in higher effectiveness than Hualong directly or indirectly. Among institutions in the same consortia, relationships are strengthened by collaboration in multiple ways (which is called network multiplex in network analysis [50]), especially referral and joint training. The strengthened relationships among institutions lead to other types of collaboration and synergistic member growth. Finally, contextual factors have accounted for the differences between the two systems [54,55]. Leichsenring [56] and Mur-Veeman [47] proposed that poorly developed integrated care likely goes hand in hand with weak primary care and community service. If the acute care sector dominates or primary care hasn’t met the basic demands of people, integration is suppressed. Therefore, the different pace towards integrated health systems in the two counties is a result of system-wide contextualization. Moreover, integrated care is the ultimate aim of integration, with seamless care from diagnosis, through treatment, rehabilitation and health promotion [57]. An integrated health system is a prerequisite for integrated care. The different paces of adoption of integrated care among different countries result in part from different national health system contexts.

## Limitation

The structures analyzed in this paper are mainly focused on health service delivery, including three of the six blocks of health systems [58], namely health service delivery, workforce and information. System structures of financing, governance, medical products and technology provision also have influence on whole system effectiveness. Future investigation could study the interaction of different structures and their joint impacts on network effectiveness. Network effectiveness in this paper is assessed from the perspective of the whole network. However, further research could use ego analysis to evaluate roles and positions of key individual institution (especially county-level institutions).

As mentioned above, social network analysis provides a snapshot at one point in time. And the survey was conducted after nine months of health system reform in Huangzhong, the structure and operation of the system may not be stable. With the evolution of contextualization, structure, and effectiveness of health care systems, network analysis would be conducted regularly to assess the dynamic nature of system development.

There are two possible biases of the study: (1) Because of the natural conditions in Qinghai province, distribution of population and economy are very uneven. We could not find a county more matching with Huangzhong from every aspect than Hualong. There may be a sampling bias, which has influence on network information. (2) The small number of patients surveyed may affect the representativeness of the sample.

## Conclusions

This article has demonstrated how network analysis can be conducted in county health systems, what the structures of two systems look like and how network effectiveness is influenced by structure and contexts. It has contributed to both theory and practice. In theory, it is the first study of county health systems using social network analysis in China, which introduces viable and valuable inter-organizational network analysis into health system research. In practice, although the study was conducted in two counties, it seems reasonable to embed it into routine operations to help policy makers promote, monitor and assess health system development towards integration. The use of medical consortia in Huangzhong is only one type of integration. Future network analysis may consider alternative forms of health system contextualization and integration models. With the development of integrated health care systems, it is possible to achieve higher efficiency at lower cost at the system-level, so supporting universal health coverage at the country-level.

As suggested by Provan [50], it is easy to agree on the principle of collaboration, but it is less easy to build an integrated network of many institutions each with a stake in practice.
